# Pyrene-Fullerene C_60_ Dyads as Light-Harvesting Antennas

**DOI:** 10.3390/molecules19010352

**Published:** 2013-12-30

**Authors:** Gerardo Zaragoza-Galán, Jesús Ortíz-Palacios, Bianca X. Valderrama, Alejandro A. Camacho-Dávila, David Chávez-Flores, Víctor H. Ramos-Sánchez, Ernesto Rivera

**Affiliations:** 1 Facultad de Ciencia Químicas, Universidad Autónoma de Chihuahua, Campus Universitario #2, Apartado Postal 669, Chihuahua 31125, Mexico; E-Mails: acamach@uach.mx (A.A.C.-D.); dchavez@uach.mx (D.C.-F.); vramos@uach.mx (V.H.R.-S.); 2 Instituto de Investigaciones en Materiales, Universidad Nacional Autónoma de México, Ciudad Universitaria, Mexico D.F. 04510, Mexico; E-Mails: jesus2ortiz@yahoo.com.mx (J.O.-P.); biancaxvg@msn.com (B.X.V.)

**Keywords:** pyrene, excimers, fullerene, fluorescence, energy transfer, photovoltaics

## Abstract

A series of pyrene-fullerene C_60_ dyads bearing pyrene units (PyFC_12_, PyFPy, Py_2_FC_12_ and PyFN) were synthesized and characterized. Their optical properties were studied by absorption and fluorescence spectroscopies. Dyads were designed in this way because the pyrene moeities act as light-harvesting molecules and are able to produce “monomer” (PyFC_12_) or excimer emission (PyFPy, Py_2_FC_12_ and PyFN). The fluorescence spectra of the dyads exhibited a significant decrease in the amount of pyrene monomer and excimer emission, without the appearance of a new emission band due to fullerene C_60_. The pyrene fluorescence quenching was found to be almost quantitative, ranging between 96%–99% depending on the construct, which is an indication that energy transfer occurred from one of the excited pyrene species to the fullerene C_60_.

## 1. Introduction

Photovoltaics based on fullerene C_60_ derivatives is one of the most active research areas in materials science due to the great performance of these molecules in emerging technologies for solar energy conversion [1,2,3,4,5,6]. However, the main disadvantage of fullerene C_60_ and other carbon allotropes is their poor solubility in organic solvents [7]. Fullerene C_60_ also mainly absorbs in the UV region of the electromagnetic spectra [7]. Since a wide absorption range (UV and visible) is desirable for organic photovoltaics [1], several strategies to covalently and non-covalently modify fullerene C_60_ have been employed. Among these, the incorporation of porphyrins [8], TTF [9,10], and azobenzenes [11] has been tested. One of the aims of any fullerene modification is to confer solubility in organic solvents and to improve its absorption in the UV-Vis region without affecting the photophysical and electrochemical properties of the raw fullerene [7]. Axial chromophores attached to fullerene C_60_ also serve to produce long life charge separated species in a fullerene C_60_ cage [12,13] and to isolate fullerene from the environment [14]. Fullerene-C_60_ hybrid derivatives have a wide range of applications in other fields such as chemosensors [15], biological probes [16], senzitizers and photocatalysis [17,18,19,20,21,22,23], *etc.* In addition, testing of chemical and non-covalent modification of fullerene C_60_ can be scaled to other carbon allotropes such as carbon nanotubes (CNT) and graphene [24,25]. Despite the great variety of fullerene dyads mentioned in the literature, relatively little information about pyrene-fullerene C_60_ hybrids can be found [26,27,28,29,30,31,32]. Pyrene is a very attractive chromophore due to its intrinsic optical properties such as high quantum yield, long luorescence lifetime and its ability to form excimers [33]. Pyrene has been claimed to be “by far, the most studied fluorescent probe in macromolecules” [33] and for this reason is a very useful analytical probe to test different dynamic processes in solution which occur in the pyrene lifetime regime [34,35,36,37]. Other interesting and emerging qualities of pyrene are its application as an antenna chromophore in dyads for solar energy conversion applications and as exfoliation agent for CNT and graphene dispersions [38].

Our research group has explored the photophysical properties of some pyrene model compounds [39]. We have also incorporated pyrene into several macromolecular architectures in order to design functional materials with interesting optical behaviors [40,41]. In our interest to study pyrene dynamics in macromolecules, in a previous work, we studied the photophysical properties of a new family of dendronized porphyrins labelled with pyrene units [42]. Thus, we reported a very efficient Fluorescence Resonance Energy Transfer (FRET) process from the excited state pyrene units to the ground state porphyrin. Taking advantage of the photophysical characteristics of this chromophore we were able to determine the quantitative quenching of fluorescence due to FRET, but we also obtained valuable information about pyrene dynamics, namely, the precluded formation of pyrene excimers due to the most competitive FRET phenomenon with higher rate constant. However, as dendron generation increases the excimer formation mechanism is favored and becomes a competitive process with FRET. Therefore, we concluded that pyrene acts as an efficient donor for FRET when it is combined with a porphyrin. Furthermore, pyrene can donate energy in a dual mode: monomer or excimer emission, depending on the molecular structure of the macromolecule [42].

In this work, we extended the study of pyrene excimer formation to hybrid materials that exhibit a complex photophysical behavior. It is worth pointing out that pyrene and fullerene C_60_ show a very small spectral overlap between pyrene emission and fullerene absorption, which precludes the possibility of an efficient FRET from pyrene to fullerene C_60_. Nevertheless, it was observed that efficient photoinduced energy transfer takes place [27,28]. The main goal of this project is the synthesis and characterization of novel pyrene-fullerene C_60_ dyads as well as the study of the resulting energy transfer process by means of steady-state fluorescence.

## 2. Results and Discussion

### 2.1. Synthesis of Pyrene-Fullerene C_60_ Dyads

**Scheme 1:**
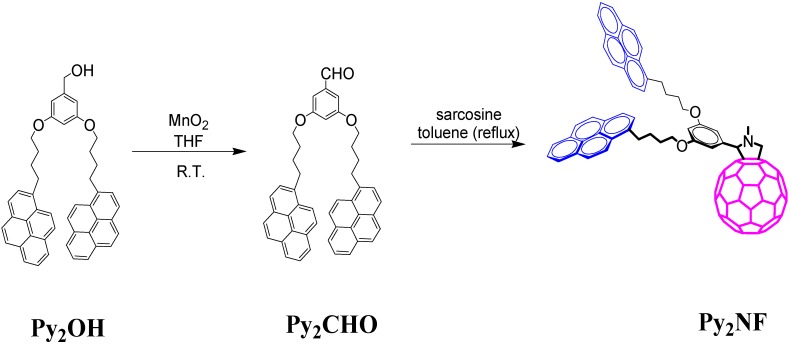
Synthesis of pyrene-labeled fulleropyrrolidine dyad.

**Figure 1:**
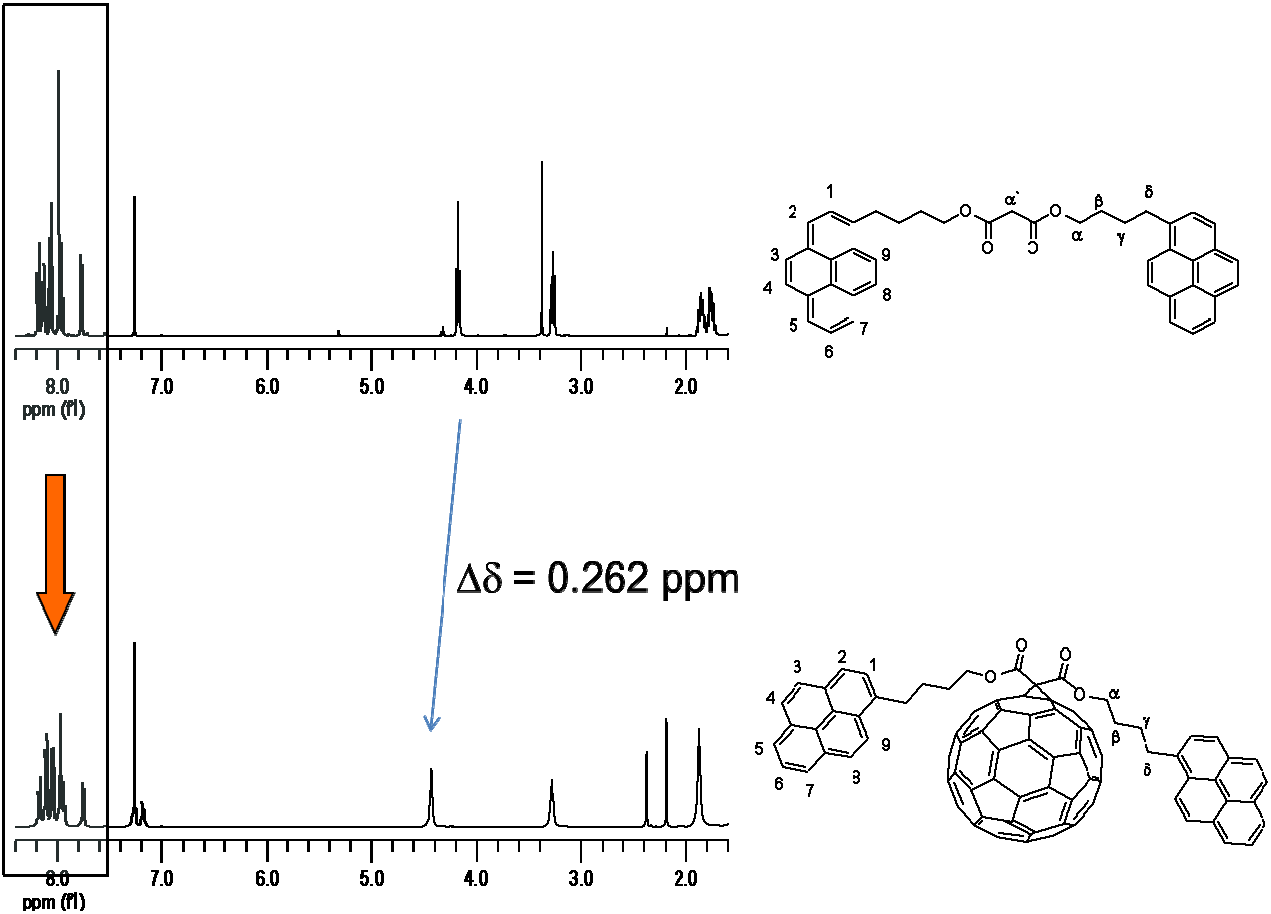
^1^H-NMR in CDCl_3_ of PyMPy bismalonate and PyFPy dyad.

Fulleropyrrolidine Py_2_FN was synthesized by reacting 3,5-bis(4-(pyren-1-yl)butoxy)benzaldehyde and N-methylglycine (sarcosine) in refluxing toluene (Scheme 1). Py_2_NF possesses one fullerene unit and two axially attached pyrene units. The non-functionalized homologue compound Py_2_OH has been reported [42] to very efficiently produce pyrene-type excimer emission via diffusion of pyrenes in the media, as it was demonstrated by steady-state fluorescence and excitation spectra. Inclusion of the fullerene C_60_ moiety in the Py_2_NF derivative is supposed to generate different pyrene fluorescence deactivation pathways which are able to compete with excimer formation. The aim of this work was to elucidate at which degree the incorporation of fullerene C_60_ can preclude excimer formation phenomena. Fulleropyrrolidine was characterized by NMR spectroscopy (Figure 1) and the structural elucidation was confirmed by means of mass spectrometry using the MALDI-TOF technique with α-cyano-4-hydro-cinnamic acid as matrix. The mass spectrometry analysis showed a peak corresponding to the molecular ion M^+^ with *m/z* = 1,398.024, which matches well with the calculated value (M^+^ *m/z* = 1398.51).

**Scheme 2:**
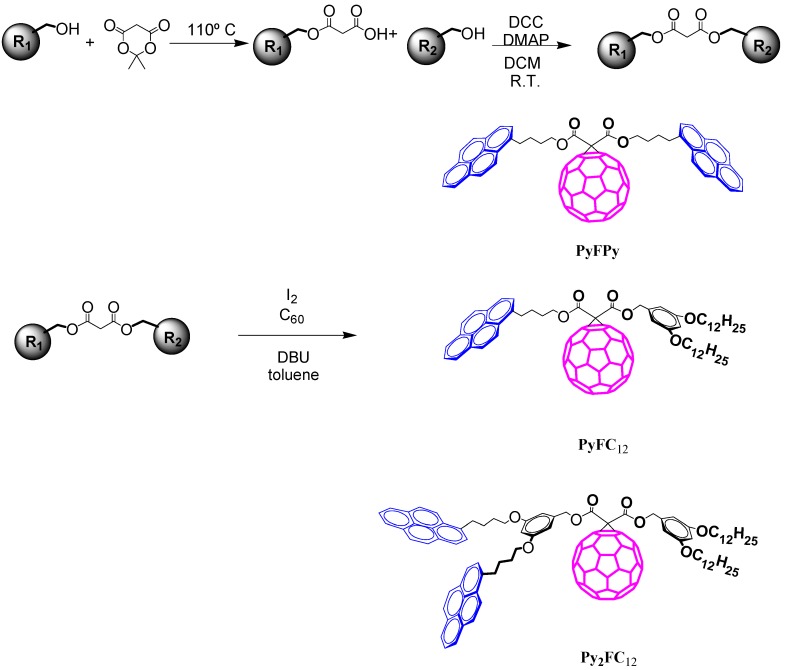
Synthetic pathway for pyrene-labeled bismalonate adducts.

Compounds PyMPy, PyMC_12_ and Py_2_MC_12_ were synthesized by employing the Meldrum’s acid methodology as reported in earlier publications [43]. From the reaction of 1-pyrenebutanol and Meldrum’s acid we were able to obtain the precursor acid PyMCOOH. Further esterification of PyMCOOH with 3,5-bis-dodecylbenzyl alcohol and 1-pyrenebutanol via DCC-coupling afforded the molecules PyMC_12 _and bismalonate PyMPy bearing one pyrene unit and two pyrene units, respectively. Esterification of the previously reported monomalonyl ester C_12_MCOOH with 3,5-bis(4-(pyren-1-yl)butoxy)benzyl alcohol using DCC as a coupling agent afforded the compounds Py_2_MC_12_, PyMPy and Py_2_MC_12_ bearing pyrene ends and able to form excimers as has been reported for similar pyrene end-labels having short chains [34,35,36,37] (Scheme 2). Pyrene-fullerene C_60_ dyads were prepared using the Bingel-Hirsch reaction [43] between bismalonate molecules PyMPy, PyMC_12_ and Py_2_MC_12_, and fullerene C_60_ in toluene. In so doing, PyFPy, PyFC_12_ and Py_2_FC_12 _dyads were obtained.

It is worth noticing that the PyFPy, Py2NF and Py_2_FC_12 _dyads are able to form excimers via diffusion of pyrene units, however, in the PyFPy molecule pyrene diffusion is blocked by the substitution of the protons in the malonate bridge by a fullerene C_60_ cage as it was reported in the literature [26]. In contrast, compounds Py_2_NF and Py_2_FC_12_ readily form excimers, since the fullerene C_60_ unit is not in the diffusive pathway of the pyrene moieties. By tuning the excimer formation mechanism via well-controlled structural characteristics of the dyads, we can study the dynamics of excimer formation and also the possible deactivation pathways of this process, namely FRET or charge transfer phenomena.

### 2.2. Absorption Spectra of Pyrene-Donor Molecules and Pyrene-C_60_ Dyads

**Figure 2:**
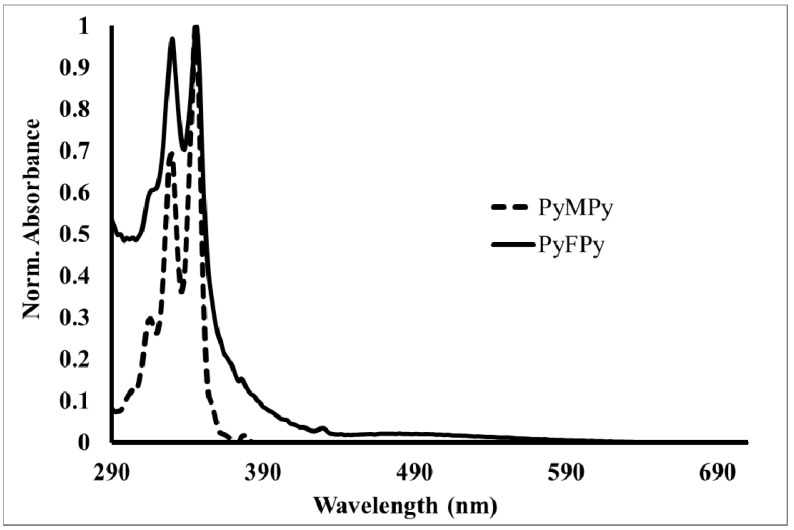
Absorption spectra of pyrene-labeled donor molecules (PyMPy; dotted line) and pyrene-fullerene dyad (PyFPy; solid line).

Absorption spectra in toluene solution of PyMPy, PyMC_12_ and Py_2_MC_12_ (Figure 2) exhibited the typical absorption bands of pyrene-derivatized compounds with a maximum absorption band at λ = 346 nm due to the S_0_→S_2_ transition. According to Duhamel *et al.* [34,35,36,37] if the peak-to-valley ratio at the S_0_→S_2_ transition band shows a value lower than 2.5 there is pyrene preassociation in the ground state which leads to the formation of static excimers. Inspection of the electronic spectra shows that the numerical values of peak-to-valley ratio for the S_0_→S_2_ transition (A_346_/A_336_) of PyMPy, PyMC_12_ and Py_2_MC_12_ are lower than 2.5. Therefore, there is weak pyrene preassociation in the ground state for these molecules. It is important to highlight this feature because it suggests that if the fluorescence spectra shows excimer-type emission, the nature of the excimer formation would rise from pyrene diffusion in media, *i.e.*, a dynamic process [34,35,36,37]. On the other hand, absorption bands at 346 nm do not show remarkable shift respect to pyrenebutanol, used as model compound, which show that the electronic properties of pyrene moieties in PyMPy and PyMC_12 _remain alike to those of pyrenebutanol.

**Figure 3:**
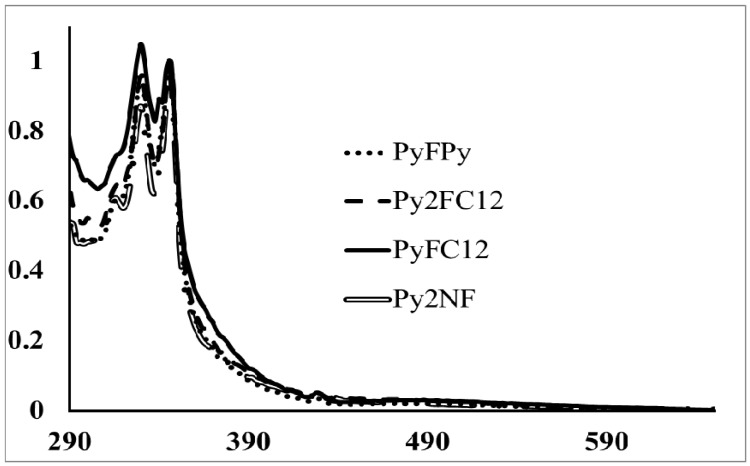
Absorption spectra of pyrene-fullerene dyads.

The electronic spectra of dyads PyFPy, PyFC_12_ and Py_2_FC_12_ (Figure 3) in toluene solution exhibited similar spectral features to those of pyrenebutanol on the top of the fullerene C_60_ absorption background. PyFPy dyad shows an intense absorption band at 330 nm due to the high extinction coefficient of the fullerene C_60_ cage, followed by a band at 346 nm attributed to the pyrene moieties as well as an extended absorption in the visible region from 400–700 nm, which is entirely attributed to fullerene C_60_ cage. It is noticeable the extremely low extinction coefficient of the absorption at 682 nm due to forbidden S_0_→S_1_ transition of fullerene. In contrast, in PyFC_12_ and Py_2_FC_12 _the pyrene bands are more resolved due to the higher pyrene local concentration in the molecules. These compounds showed similar features as those discussed above. A unique pyrene unit is present in PyFC_12_, this precludes the possibility of intramolecular excimer formation. On the contrary, PyFPy and Py_2_FC_12_ which contain two pyrene units in their structure can lead to excimer formation. However, in PyFPy pyrene interactions are blocked by the presence of the fullerene moiety. Surprisingly, excimer formation is still possible in Py_2_FC_12_ since the fullerene unit is not in the middle of the pyrene diffusion pathway. It is remarkable that the peak shifts of the fullerene and pyrene transitions were not noticeable, suggesting that there is no remarkable electronic interaction between both chromophores, as discussed earlier; this trend was also confirmed by NMR spectroscopy. 

### 2.3. Steady-State Fluorescence of Pyrene-Donor Molecules and Pyrene-C_60_ Dyads

**Figure 4:**
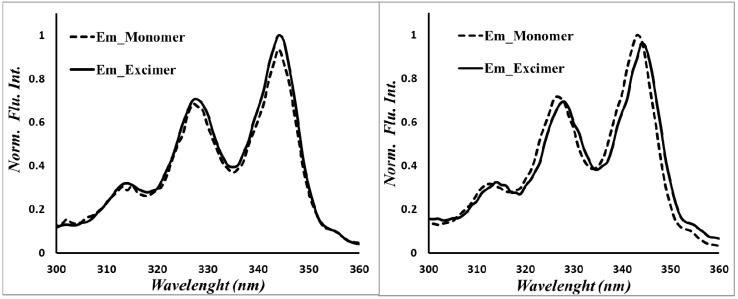
Excitation spectra of Py_2_OH (left) and PyMPy (right) recorded at λ_F_ monomer = 398 and λ_F_ excimer = 478 nm.

Fluorescence spectra of donor molecules PyMPy, pyrenebutanol and Py_2_OH were acquired in toluene solution at room temperature, exciting at 344 nm. The steady-state emission spectra of PyMPy and Py_2_OH show the characteristic pyrene monomer emission at 370 nm, followed by a broad excimer emission band centered at 478 nm. For PyOH, the fluorescence spectra show a uniquely monomer emission at 370 nm. The excimer emission intensity (*I*_E_) relative to that of the monomer emission (*I*_M_), namely the *I*_E_/*I*_M_ ratio, increases with the number of pyrene groups in the construct from 0.6 for Py_2_OH and 2 for PyMPy. The compound PyMPy showed significantly higher excimer emission and lower monomer emission than Py_2_OH, even if they present the same local pyrene concentration. This is because PyMPy has a more flexible backbone and shows a better ability to diffuse in the media to efficiently form excimers. The nature of the excimers for Py_2_OH and 2 for PyMPy appears to be dynamic, since the absorption spectra overlap that of 1-pyrenebutanol. In addition, the excitation spectra of the pyrene monomer and excimer acquired at 398 and 478 nm, respectively, overlap for both constructs (Figure 4). The nature of the excimer formation was elucidated by excitation spectra of the donor molecules PyMPy and Py_2_OH. Analysis of the peak-to-valley ratio of S_0_→S_2_ transition in the electronic spectra suggested that the pyrene units are not pre-associated in the ground state. Thus, the excimers are dynamic in nature. This was confirmed by excitation spectra of the donor PyMPy and Py_2_OH recorded at the monomer (390 nm) and excimer (470 nm) emission. Since excitation spectra were identical in both samples for the monomer (390 nm) and excimer (470 nm) emission the dynamic nature of the excimers has been confirmed [34,35,36,37].

**Figure 5:**
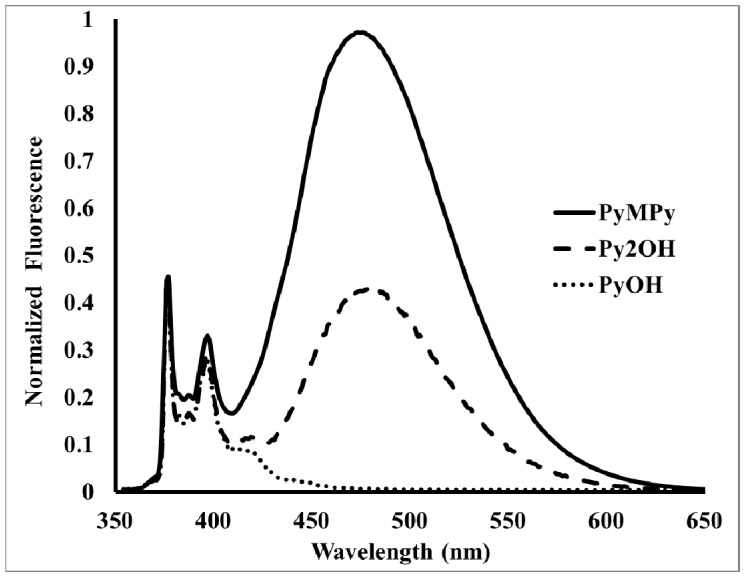
Emission spectra of pyrene-labeled donor molecules (λ_exc_ = 344 nm).

**Figure 6:**
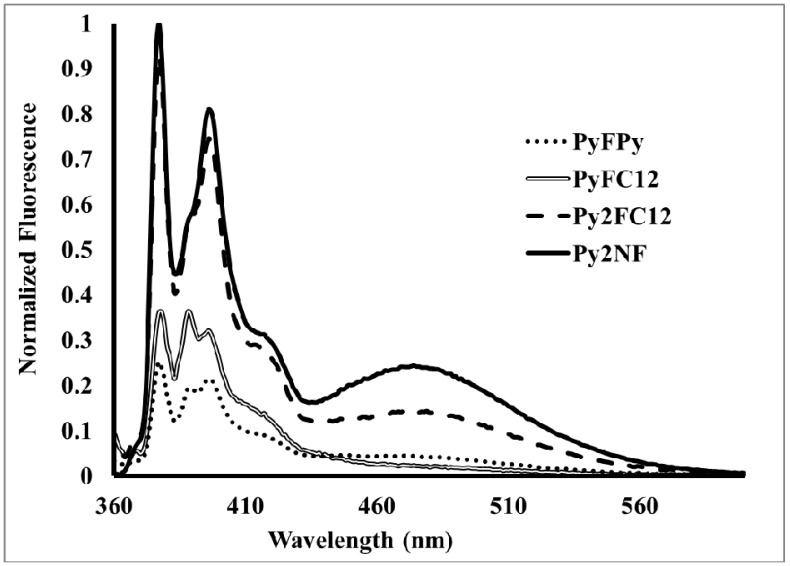
Emission spectra of pyrene-labeled dyads (λ_exc_ = 344 nm).

The fluorescence spectra of PyFC12, Py2FC12, PyFPy and Py2NF were acquired in toluene solution at room temperature exciting at 344 nm. Figure 5 shows the emission spectra of the pyrene-labeled constructs. They exhibited typical monomer-type pyrene fluorescence at 390 nm. Since fluorescence emission of the dyads is low, the emission profile is altered by the dispersion peaks of the solvent at the excitation wavelength. Previously, we reported a similar behavior in pyrene dendronized porphyrins [42]. However, in the present study, accurate fluorescence profiles were not retrieved after solvent correction, due to a possible photobleaching of the pyrene moieties after excitation. Fluorescence emission is almost quantitavely quenched in all the dyads with quenching values over 96% (Figure 6 and Table 1). It is remarkable that compounds Py_2_FC_12_ and Py_2_NF exhibited residual excimer-type emission at *ca.* 470 nm. A very small I_E_/I_M_ ratio was calculated for Py_2_FC_12_ and Py_2_NF, 0.14 and 0.24, respectively. The quite low excimer emission would rise from small fraction of pyrenes diffusing in media and also from those pyrene units whose fluorescence are not deactivated by fullerene units. It is interesting to remark the fact that the donor molecule PyMPy exhibited a high I_E_/I_M_ ratio which suggests a very efficient dynamic excimer formation. In contrast, PyFPy does not show any excimer emission, due the presence of the fullerene unit which prevents the pyrene diffusion pathway. As could be expected, PyFC_12 _does not exhibit any excimer emission.

**Table 1:** Relative quatum yield of the studied compounds.

Compound	Relative Quantum Yield ^a^	% Quenching
1-Pyrenbutanol	1 (Donor)	-------
PyFC12	0.01 (Dyad)	99%
PyMPy	1 (Donor)	-------
PyFPy	0.01 (Dyad)	99%
Py2OH	1 (Donor)	------
Py2FC12	0.01 (Dyad)	99%
Py2NF	0.04 (Dyad)	96%

^a^ Measured in degassed toluene solution exciting at λ = 344 nm. Relative QY of the dyads, with respect to the donor molecules (1-pyrenebutanol, PyMPy and Py_2_OH) in the range of 360 nm–560 nm.

After excitation at 344 nm in toluene solution, where absorption of pyrene and fullerene C_60_ is observed, the pyrene emission at 390 nm is almost quantitatively quenched and no emission from the fullerene C_60_ moiety was detected, even at 708 nm where the derivatized-fullerene emission usually appears. We carried out a comparison of the fluorescence of the fullerene C_60_ (quantum yield lower than 0.1%) [21] and the obtained dyads and we observed that the emission of the latter is significantly lower. This dramatic quenching in toluene, which is a relatively low dielectric constant solvent, indicates that the fluorescence deactivation pathway is mainly due to a fast energy transfer from excited pyrene to fullerene C_60_. A similar behavior was observed in a similar fullerene C_60_ dendritic molecule bearing 8 pyrene moieties previously reported in the literature [32]. The geometry of the molecule allows the π-π interactions of the pyrene moieties with the fullerene cage thereby favoring energy transfer mechanism.

## 3. Experimental

### 3.1. General

All the reagents involved in the synthesis were purchased from Aldrich (Mexico D.F.) and employed as received. The solvents used in the reactions were purified by simple distillation. 3,5-bis-Dodecylbenzyl alcohol [43], monomalonyl ester C_12_MCOOH [43] and 3,5-bis(4-(pyren-1-yl)butoxy)-benzyl alcohol [42] were obtained according to previously reported procedures. FTIR spectra of the intermediates, dendrons and pyrene-fullerene dendritic systems were recorded on a Spectrum 100 spectrometer (Perkin Elmer, Waltham, MA, USA) in solid state. ^1^H and ^13^C-NMR spectra of these compounds in CDCl_3_ solution were recorded at room temperature on a Bruker Avance 400 MHz spectrometer (Billerica, MA, USA), operating at 400 MHz and 100 MHz for ^1^H and ^13^C, respectively. MALDI-TOF measurements were obtained in a Bruker-Microflex spectrometer. For UV-Vis and fluorescence spectroscopies, tetrahydrofuran (THF) was purchased from Aldrich (spectrophotometric grade). Prior to use, the solvent was checked for spurious emission in the region of interest and found to be satisfactory. The absorption spectra of the final compounds in solution were recorded on a Varian Cary 1 Bio UV/vis spectrophotometer (Palo Alto, CA, USA) using 1 cm quartz cells and solute concentrations of (1 × 10^−6^–3 × 10^−6^) M for all compounds. It has been verified that the Beer-Lambert law applies for the used concentrations. Fluorescence spectra corrected for the emission detection were recorded on a Photon Technology International LS-100 steady-state fluorimeter (New Jersey, NJ, USA) having a continuous Ushio UXL-75Xe Xenon arc lamp (New Jersey, NJ, USA) and a PTI 814 photomultiplier detection system. Each solution was excited at 344 nm using a 1 cm quartz cell. For all compounds, a pyrene concentration of less than 1.25 × 10^−6^ M was used to ensure that the solutions would have an absorbance of 0.05 at 344 nm and to avoid any inner filter effect [44].

### 3.2. Synthesis of 3,5-Bis(4-(pyren-1-yl)butoxy) Benzaldehyde

To a solution of 3,5-bis(4-(pyren-1-yl)butoxy)benzyl alcohol (0.501 g, 0.767 mmol) in THF (30 mL), MnO_2_ (0.806 g, 9.274 mmol) was added and the reaction mixture was stirred at room temperature under argon atmosphere for 16 h. After this time, the solution was filtered over a Celite plug, and the solvent was evaporated under reduced pressure. The crude product was recrystallized from CH_2_Cl_2_-hexanes and dried under vacuum. The final product was obtained as a white solid (0.429 g, 0.659 mmol). Yield: 85.8%. ^1^H-NMR (CDCl_3_): 9.83 (Hα', s, 1H), 8.28–7.85 (Har-py, m, 18H), 6.93 (Ho, d, *J* = 2.25 Hz, 2H), 6.63 (Hp, t, *J* = 2.23 Hz, 1H), 3.97 (Hα, t, *J* = 7.57 Hz, 4H), 2.09–1.88 (Hβ and Hγ, m, 9H).

### 3.3. Synthesis of Bis[4-pyrene-1-butoxy]malonyl Ester

A mixture of 1-pyrenebutanol (0.3 g, 1.09 mmol) and Meldrum’s acid (0.137 g, 1.2 mmol) was heated at 120 °C for 5 h under continuous stirring and under an argon atmosphere. The crude product was purified by chromatographic column of SiO_2_. Two products were isolated by column chromatography on SiO_2_: (a) [4-pyrene-1-butoxy]monomalonyl acid: (eluent DCM/MeOH 98/2). White solid (0.216 g, 0.601 mmol). Yield 55%. ^1^H-NMR (CDCl_3_): 8.25–7.81 (HAr-py, m, 9H), 3.39 (Hα, s, 2H), 3.36 (Hδ', m, 2H), 1.93-1.82 (Hβ' and γ', m, 4H); (b) bis[4-pyrene-1-butoxy]malonyl ester: (eluent hexanes/DCM 80/20). White solid (0.134 g, 0.218 mmol). Yield: 40%.^1^H-NMR (CDCl_3_): 8.21–7.75 (HAr-py, m, 18H), 4.19, 4.16, 4.13 (Hα', t, *J* = 6.2 Hz, 4H), 3.37 (Hα, s, 2H), 3.3, 3.26, 3.23 (Hδ', t, *J* = 7.2 Hz, 4H), 1.84–1.77 (Hβ'and γ', m, 8H).

### 3.4. General Procedure for DCC Esterification

DCC (2.2 equiv) was added to a stirred solution of monomalonyl acid (1 equiv), the appropriate alcohol (2 equiv), and DMAP (0.5 equiv) in CH_2_Cl_2_ at 0 °C. After 12 h, the mixture was allowed to reach room temperature (about 1 h), then it was filtered and evaporated at reduced pressure. The crude product was then purified as outlined below.

#### 3.4.1. Synthesis of [3,5-Bis(dodecyl)-benzyl]-[4-(pyren-1-yl)butoxy]malonyl Ester

From DCC (0.0806 g), [3,5-bis(dodecyl)benzyl]malonic acid (100 mg), 1-pyrenebutanol (0.0975 g), DMAP (0.0109 g). The crude was purified by SiO_2_ column chromatography, eluting with DCM/hexanes (30/70).The final product was obtained as a white solid (0.087 mg, 0.106 mmol). Yield: 60%.^ 1^H-NMR (CDCl_3_): 8.23–8.03 (HAr-py, m, 9H), 6.44 (Ho, m, 2H), 6.38 (Hp, m, 1H), 5.08 (Hα', s, 2H), 4.23 (Hα'', m, 2H), 3.88 (Hβ1, m, 4H), 3.44 (Hα, s, 2H), 3.36 (Hδ''', m, 2H), 1.85 (Hβ'' and γ'', m, 4H), 1.73 (Hβ2, m, 4H), 1.25 (Hβ3 and Hβ11, m, 36H), 0.88 (Hβ12, m, 6H).

#### 3.4.2. Synthesis of [3,5-Bis(dodecyl)-benzyl]-[3,5-bis(4-(pyren-1-yl)butoxy)benzyl]malonyl Ester

From DCC (0.806 g), [3,5-bis(dodecyl)-benzyl]malonic acid (0.1 g), 3,5-bis(4-(pyren-1-yl)butoxy)-benzyl alcohol (0.232 g), DMAP (10.85 mg). The crude was purified by SiO_2_ column chromatography, eluting with DCM/hexanes (50/50).The final product was obtained as a yellow oily-solid (0.097 g, 0.081 mmol). Yield: 46%.^ 1^H-NMR (CDCl_3_): 8.30–7.85 (HAr-py, m, 18H), 6.49 (Ho', m, 2H), 6.45 (Ho, m, 2H), 6.40 (Hp and Hp', m, 2H), 5.10 (Hα and Hα', m, 4H), 3.97 (Hα1', t, *J* = 6.2 Hz, 4H), 3.90, 3.87, 3.83 (Hα1, t, *J* = 6.4 Hz, 4H), 3.49 (Hα, s, 2H), 3.412 (Hα4', t, *J* =7.4 Hz, 4H), 1.97 (Hα2' and Hα3', m, 8H), 1.72 (Hα2, m, 4H), 1.26 (Hα3 and Hα11, m, 36H), 0.89 (Hα12, m, 6H).

### 3.5. General Procedure for Fullerene C_60_ Dyads by Bingel-Hirsch Reaction

DBU (5 equiv) was added to a stirred solution of C_60_ (1 equiv), I_2_ (2.5 equiv) and the appropriate bismalonate (1.1 equiv) in toluene. The solution was stirred for 24 h, then filtered through a short plug of SiO_2_, eluting first with toluene (to remove unreacted fullerene C_60_) and then with DCM:hexanes to yield the corresponding product.

#### 3.5.1. Synthesis of [3,5-Bis(dodecyl)benzyl]-[4-(pyren-1-yl)butoxy]malonyl Ester-Fullerene C_60_

From DBU (0.443 mmol, 66.8 µL), fullerene C_60_ (0.088 mmol, 0.064 g), I_2_ (0.222 mmol, 0.056 g), [3,5-bis(dodecyl)benzyl]-[4-(pyren-1-yl)butoxy]malonyl ester (0.097 mmol, 0.080 g), toluene (100 mL). Column chromatography (SiO_2_): toluene (100%) and then DCM/hexanes (50/50). The final product was obtained as a brown solid (0.058 g, 0.038 mmol). Yield: 39%.^ 1^H-NMR (CDCl_3_): 8.26–7.81 (HAr-py, m, 9H), 6.54 (Ho, m, 2H), 6.39 (Hp, m, 1H), 5.36 (Hα, s, 2H), 4.56 (Hα', s, 2H), 3.88, 3.85, 3.81 (Hβ1, t, *J* = 6.2 Hz, 4H), 3.38 (Hδ', m, 2H), 1.98 (Hβ' and γ', m, 4H), 1.70 (Hβ2, m, 4H), 1.24 (Hβ3 and Hβ11, m, 36H), 0.87 (Hβ12, m, 6H). MALDI-TOF MS calculated for C_114_H_72_O_6_ [M]^+^ *m/z* = 1,537.79; found: [M]^+^ *m/z* = 1,538.209.

#### 3.5.2. Synthesis of Bis[4-pyrene-1-butoxy]malonyl Ester-Fullerene C_60_

From DBU (0.737 mmol, 0.11 mL), fullerene C_60_ (0.147 mmol, 0.106 g), I_2_ (0.093 g, 0.368 mmol), bis[4-(pyren-1-yl)butoxy]malonyl ester (0.1621 mmol, 100 mg), toluene (110 mL). Column chromatography (SiO_2_): toluene (100%) and then DCM/hexanes (50/50). The final product was obtained as a brown solid (0.058 g, 0.044 mmol). Yield: 27%. ^1^H-NMR (CDCl_3_): 8.21–7.75 (HAr-py, m, 18H), 4.44 (Hα, m, 4H), 3.29 (Hδ, m, 4H), 1.88 (Hβ and γ, m, 8H). MALDI-TOF MS. Calculated for C_103_H_34_O_4_ [M]^+^ *m/z* =1,335.37. Found: [M]^+^ *m/z* = 1,335.851.

#### 3.5.3. Synthesis of [3,5-Bis(dodecyl)-benzyl]-[3,5-bis(4-(pyren-1-yl)butoxy)-benzyl]malonyl Ester-Fullerene C_60_

From DBU (0.335 mmol, 50 µL), fullerene C_60_ (0.067 mmol, 0.048 g), I_2_ (0.167 mmol, 0.042 g), [3,5-bis(dodecyl)benzyl]-[3,5-bis(4-(pyren-1-yl)butoxy)-benzyl]malonyl ester (0.075 mmol, 0.09 g), and toluene (50 mL). Column chromatography (SiO_2_): toluene (100%) and then DCM/hexanes (50/50). The final product was obtained as a brown solid (41 mg, 0.0214 mmol). Yield: 32%. ^1^H-NMR (CDCl_3_): 8.23-7.81 (HAr-py, m, 18H), 6.55 (Ho' and Ho, m, 4H), 6.40-6.36 (Hp and Hp', m, 2H), 5.39 (Hα' and Hα'', m, 4H), 3.89 (Hα1 and Hα1', m, 8H), 3.35 (Hα4', m, 4H), 1.93 (Hα2' and Hα3', m, 8H), 1.76 (Hα 4.2, m, 4H), 1.24 (Hα3 and Hα11, m, 36H), 0.88 (Hα12, m, 6H). MALDI-TOF-MS. Calculated for C_141_H_94_O_8_ [M]^+^ *m/z* = 1,916.25. Found: [M]^+^ *m/z* = 1,916.612.

#### 3.5.4. Synthesis of Fulleropyrrolidine

To a solution of fullerene C_60_ (0.1 g, 0.14 mmol) in toluene (100 mL), N-methylglycine (0.250 g, 0.28 mmol) and 3,5-bis(4-(pyren-1-yl)butoxy)benzaldehyde (0.182 g, 0.28 mmol) were added. The mixture was refluxed overnight under argon atmosphere. Then, the reaction was allowed to reach room temperature and the solvent evaporated under reduced pressure. The crude was purified by column chromatography on SiO_2_, first eluting with toluene to remove the unreacted fullerene C_60_ and then with DCM/hexanes (50/50). The final product was obtained as a brown solid (0.148 g, 0.106 mmol). Yield: 38%. ^1^H-NMR (CDCl_3_): 8.25–7.81 (HAr-py and Ho inside, m, 20H), 6.42 (Hp, m, 1H), 4.89, 4.85 (H α'2, d, *J* = 9.2 Hz, 1H), 4.73 (Hα1, s, 1H), 4.16, 4.11 (H α2, d, *J* = 9.6 Hz, 1H), 4.02 (H α, m, 4H), 3.34 (Hδ, m, 4H), 2.77 (HA, s, 3H), 1.93 (Hβ and γ, m, 8H). MALDI-TOF MS. Calculated for C_109_H_43_NO_2_ [M]^+^ *m/z* = 1,398.51. Found: [M]^+^ *m/z* = 1,398.024.

## 4. Conclusions

The optical properties of pyrene model molecules PyMPy and Py_2_OH were studied by absorption spectroscopy and steady-state fluorescence. PyMPy and Py_2_OH form excimers very efficiently as it was demonstrated by steady-state fluorescence. Pyrene excimer formation is dynamic in nature which implies a diffusion pathway. In this work, pyrene was appended to a fullerene moiety cage using different motifs that allowed pyrene excimer formation via intermolecular diffusion. By employing this strategy absorption of the fullerene unit in the far UV region was increased. Pyrenes act as antennae molecules able to harvest UV radiation in a very efficient way. Also axially appended pyrenes are free to be used as binding motifs to disperse carbon allotropes as it has been previously reported in the literature. Furthermore, pyrene excimer diffusion was tested in this model compounds since molecular control of the pyrene dynamics was achieved by the incorporation of fullerene C_60_ in different positions. In molecules PyFPy, Py_2_FC_12_ and Py_2_NF, pyrene diffusion was possible in all cases, however, fullerene C_60_ blocked dramatically the pyrene diffusion pathway in PyFPy. This phenomenon was evident since residual pyrene excimer-type emission was precluded in PyFPy. In Py_2_FC_12_ and Py_2_NF pyrene excimer formation was favored since fullerene C_60_ did not interfere in the pyrene diffusion pathway. This was also confirmed by the increase in the residual pyrene excimer-type emission.
